# m^6^A-mediated upregulation of AC008 promotes osteoarthritis progression through the miR-328-3p‒AQP1/ANKH axis

**DOI:** 10.1038/s12276-021-00696-7

**Published:** 2021-11-04

**Authors:** Jiashu Yang, Ming Zhang, Dawei Yang, Yunfei Ma, Yuting Tang, Mengying Xing, Lingyun Li, Li Chen, Yucui Jin, Changyan Ma

**Affiliations:** 1 Jiangsu Key Laboratory of Xenotransplantation, Nanjing Medical University, Nanjing, P.R. China; 2 Department of Medical Genetics, Nanjing Medical University, Nanjing, P.R. China; 3 Department of Orthopaedic Surgery, Nanjing First Hospital, Nanjing, P.R. China; 4 Guangxi Key Laboratory of Tumor Immunology and Microenvironmental Regulation, Guilin Medical University, Guilin, P.R. China; 5 Department of Endocrinology and Metabolism, Endocrine Research Laboratory (KMEB), Odense University Hospital and University of Southern Denmark, Odense, Denmark

**Keywords:** Mechanisms of disease, Osteoarthritis, Long non-coding RNAs

## Abstract

Long noncoding RNAs (lncRNAs) have emerged as important regulators of osteoarthritis (OA), but the biological roles and clinical significance of most lncRNAs in OA are not fully understood. Microarray analysis was performed to identify differentially expressed lncRNAs, mRNAs, and miRNAs between normal and osteoarthritic cartilage. We found that AC008440.5 (abbreviated AC008), as well as AQP1 and ANKH, were highly expressed in osteoarthritic cartilage, whereas miR-328-3p was expressed at a low level in osteoarthritic cartilage. Functional assays showed that ectopic expression of AC008, AQP1, and ANKH significantly decreased chondrocyte viability and promoted chondrocyte apoptosis and extracellular matrix (ECM) degradation, whereas knockdown of AC008, AQP1, and ANKH resulted in the opposite effects. Moreover, miR-328-3p overexpression increased chondrocyte viability and attenuated chondrocyte apoptosis and ECM degradation, whereas inhibition of miR-328-3p resulted in the opposite effects. Bioinformatics analysis, RNA immunoprecipitation (RIP), and luciferase assays revealed that AC008 functioned as a competing endogenous RNA (ceRNA) to regulate miR-328-3p, which specifically targeted the AQP1 and ANKH genes. In addition, miR-328-3p significantly ameliorated MIA-induced OA, whereas AC008 accelerated OA progression in vivo. Furthermore, fat mass and obesity-associated (FTO)-mediated N6-methyladenosine demethylation downregulated AC008 transcription, while lower FTO expression led to upregulation of AC008 transcription in OA. In conclusion, our data reveal that AC008 plays a critical role in OA pathogenesis via the miR-328-3p**‒**AQP1/ANKH pathway, suggesting that AC008 may be a potential therapeutic target for OA.

## Osteoarthritis: a pathway for protecting cartilage

A search for genes involved in the joint disease osteoarthritis reveals a non-coding RNA that regulates molecular pathways responsible for maintaining healthy cartilage. The genome encodes many such RNAs, which control the activity of protein-coding genes via diverse mechanisms. Researchers led by Changyan Ma and Yucui Jin at Nanjing Medical University in China recently determined that one such short RNA, known as AC008, is notably over-expressed in osteoarthritic cartilage compared with healthy tissue. They showed that AC008 impairs the survival of cartilage-forming chondrocytes and leads to degradation of the extracellular matrix surrounding them. The researchers also identified two proteins regulated by AC008 that directly contribute to this process of chondrocyte loss, and uncovered the mechanism by which this regulation occurs. This pathway could thus offer potentially useful new targets for osteoarthritis drug development.

## Introduction

Osteoarthritis (OA) is an age-related or posttraumatic degenerative joint disease^1^ that is characterized by extracellular matrix (ECM) degradation, chondrocyte hypertrophy, and apoptosis^2–4^. Patients with OA experience many problems, such as joint pain, temporary stiffness, periarticular tenderness, swelling, and limited joint function, which may lead to difficulty with activities of daily living^5^. Current treatments for OA are limited to pain suppression and alleviation of inflammation, but these measures do not modify disease progression^6^. Therefore, a better knowledge of the molecular mechanisms that underlie the development and progression of OA is needed.

Noncoding RNAs are a class of RNA molecules that lack protein-coding potential. Based on the nucleotide sequence length, noncoding RNAs are classified as small noncoding RNAs (18–200 nt) and long noncoding RNAs (lncRNAs, >200 nt)^7,8^. MicroRNAs (miRNAs), a class of small noncoding RNAs ~22 nucleotides in length, negatively regulate target gene expression either by inhibiting mRNA translation or by promoting mRNA degradation^9^. miRNAs have been well recognized to play important roles in OA pathogenesis. For example, two homologous miRNAs, miR-204 and miR-211, maintain joint homeostasis to suppress OA pathogenesis by targeting RUNX2^10^. miR-141/200c promotes OA development by targeting SIRT1, thereby activating the IL-6/STAT3 pathway^11^. A growing body of evidence indicates that dysregulation of lncRNAs is involved in the progression of OA by affecting various essential cellular features of chondrocytes, such as their proliferation, apoptosis, inflammation, and ability to contribute to ECM degradation^12,13^. For example, HOTAIR aggravates ECM degradation and chondrocyte apoptosis by sponging miR-17-5p and upregulating FUT2 expression^14^. LncRNA-XIST promotes OA progression by regulating chondrocyte viability, chondrocyte apoptosis, and ECM degradation. Mechanistically, LncRNA-XIST functions as a competing endogenous RNA (ceRNA) by competing for binding with miR-149-5p to regulate DNMT3A expression^15^. LncRNA-CRNDE regulates chondrogenic differentiation of bone marrow mesenchymal stem cells and promotes cartilage repair in OA by binding with SIRT1, thus increasing its protein stability^16^. However, due to the large number of lncRNAs and their complex mechanisms of action, studies on lncRNAs in OA are still very limited.

In the present study, we performed microarray analysis to identify differentially expressed lncRNAs, mRNAs, and miRNAs between normal and osteoarthritic cartilage. We identified a novel lncRNA, AC008440.5 (abbreviated AC008), and found that this lncRNA, as well as AQP1 and ANKH, were highly expressed in osteoarthritic cartilage, whereas miR-328-3p was expressed at low levels in osteoarthritic cartilage. Functional assays showed that ectopic expression of AC008, AQP1, and ANKH significantly decreased chondrocyte viability and promoted chondrocyte apoptosis and ECM degradation, whereas knockdown of AC008, AQP1, and ANKH resulted in the opposite effects. Moreover, miR-328-3p overexpression increased chondrocyte viability and attenuated chondrocyte apoptosis and ECM degradation, whereas inhibition of miR-328-3p exerted the opposite effects. Mechanistically, we found that AC008 acted as a ceRNA to sponge miR-328-3p and then upregulate the expression of its target genes AQP1 and ANKH. Additionally, in vivo, miR-328-3p significantly ameliorated MIA-induced OA, whereas AC008 accelerated OA progression. Furthermore, we demonstrated that fat mass and obesity-associated (FTO)-dependent N6-methyladenosine (m^6^A) demethylation was responsible for the upregulation of AC008 in OA. In conclusion, our study elucidated that AC008 plays a critical role in OA pathogenesis via the miR-328-3p‒AQP1/ANKH pathway and suggested that AC008 may be a promising therapeutic target for OA.

## Materials and methods

### Microarray analysis

Total RNAs isolated from three normal cartilage samples and three osteoarthritic cartilage samples were sent to CapitalBio Corporation (Beijing, China) for microarray analysis. A CapitalBio Technology Human LncRNA Array v4 containing probes for approximately 41,000 human lncRNAs and approximately 34,000 human mRNAs, as well as an Affymetrix miRNA 4.0 Array containing probes for 30,424 miRNAs from various species, including humans, mice, and rats, were simultaneously used to identify the differentially expressed lncRNAs, mRNAs and miRNAs between normal and osteoarthritic cartilage. Genes with *P* < 0.05 and a fold change ≥2 or ≤0.5 were considered to be significantly differentially expressed.

### Clinical samples

Human osteoarthritic cartilage was collected from OA patients undergoing total knee replacement surgery (*n* = 39, aged 49–83 years). Normal cartilage was obtained from patients with no prior medical history of OA undergoing amputation or trauma surgery (*n* = 39, aged 45–91 years). The cartilage donor information is listed in Supplementary Table 1. The study was approved by the Research Ethics Committee of Nanjing Medical University. All participants read and signed an informed consent form.

### Cell lines and cell culture

Primary human articular chondrocytes were isolated from normal articular cartilage, as previously described^17^. In brief, articular cartilage was dissected into small sections and was then digested with 0.2% type II collagenase (Sigma-Aldrich, St. Louis, MO, USA) in Dulbecco’s modified Eagle’s medium/nutrient mixture F12 (DMEM/F12) (Gibco, Carlsbad, CA, USA) at 37 °C for 16 h. The chondrocytes were then filtered through a 70 μm cell strainer and maintained in DMEM/F12 containing 10% fetal bovine serum (Gibco) in an incubator at 37 °C with 5% CO_2_. Primary chondrocytes at passage 2 were used for subsequent experiments. HEK293T cells were purchased from the American Type Culture Collection (Manassas, VA, USA) and cultured in DMEM (Gibco) supplemented with 10% fetal bovine serum in a humidified atmosphere with 5% CO_2_ at 37 °C.

### Plasmid construction, RNAi, and cell transfection

The AC008 overexpression plasmid was constructed by inserting the full-length AC008 sequence into the pcDNA3.1 vector (Invitrogen, Carlsbad, CA, USA). The AQP1, ANKH, and FTO overexpression plasmids and the empty vector were purchased from Sino Biological Inc. (Beijing, China). RiboBio (Guangzhou, China) chemically synthesized the miR-328-3p mimic, miR-328-3p inhibitor, and corresponding negative controls (Mimic-NC, Inhibitor-NC); the small interfering RNAs (siRNAs) against AQP1 and ANKH (siAQP1, siANKH) and negative control siRNA (siNC); and the antisense oligonucleotide (ASO) targeting AC008 (AC008 ASO) and negative control ASO (Control ASO). For the luciferase reporter assay, the wild-type AC008 sequence and the mutant AC008 sequence (in which only the putative miR-328-3p binding site was mutated) were synthesized and cloned into the psiCHECK-2 vector (Promega, Madison, WI, USA). The AQP1 and ANKH 3’UTRs and their respective mutant sequences (in which only the predicted miR-328-3p binding sites were mutated) were amplified and inserted into the pGL3-Promoter vector (Promega). Supplementary Table 2 lists the primers used. Cell transfection was conducted using Lipofectamine 2000 (Invitrogen, Carlsbad, CA, USA) in accordance with the manufacturer’s instructions.

### Total RNA isolation and quantitative real-time reverse transcription PCR (qRT-PCR)

Total RNA from articular cartilage and cultured cells was isolated using TRIzol reagent (Takara, Otsu, Japan). Complementary DNA was synthesized from 1 μg of total RNA using HiScript II Q Select RT SuperMix (Vazyme Biotech, Nanjing, China). The expression levels of mature miRNAs were detected by stem–loop qRT-PCR. First, the stem–loop RT primer bound to miRNA molecules and cDNA was synthesized with reverse transcriptase. Then, the RT products were amplified using a miR-328-3p-specific forward primer and a universal reverse primer. Real-time PCR was performed using FastStart Universal SYBR Green Master Mix (Roche, Indianapolis, IN, USA) in a Roche LightCycler 96 Real-Time PCR System. U6 RNA was used as an endogenous reference for miR-328-3p, and β-actin mRNA was used as an internal reference for other genes. All primers used for PCR amplification are listed in Supplementary Tables 3–5.

### Western blotting analysis

Cells were washed with phosphate-buffered saline (PBS) and harvested in radioimmunoprecipitation assay buffer (Beyotime, Haimen, China) supplemented with protease inhibitor cocktail (Roche, Indianapolis, IN, USA). Equal amounts of protein were loaded on a 10% sodium dodecyl sulfate (SDS)-polyacrylamide gel for electrophoresis and transferred to a polyvinylidene difluoride membrane (Millipore, Billerica, MA, USA). The membrane was probed with specific antibodies overnight at 4 °C and was then incubated with secondary antibodies at room temperature for 1 h. Each protein band was visualized with an ECL reagent (Millipore). Primary antibodies specific for the following proteins were used: Bax, Bcl-2, cleaved Caspase 9, cleaved PARP1, and Survivin (all from Proteintech Group, Wuhan, China), cleaved Caspase 3 (Affinity, Cincinnati, OH, USA), ANKH (ABclonal, Wuhan, China), AQP1, β-actin, MMP13, COL2A1, and ADAMTS-5 (all from Bioss, Beijing, China).

### Cell counting kit-8 (CCK-8) assay

Cell viability was assessed using a CCK-8 assay (Yeasen, Shanghai, China). Cells were seeded in 96-well plates at an initial density of 4 × 10^3^ cells per well. On Days 0–4, cells were treated with CCK-8 reagent at 37 °C for 2 h, and the optical density was measured at 450 nm with a microplate reader (BioTek, Winooski, VT, USA). All experiments were repeated three times with six replicates per experiment.

### Flow cytometric analysis

Apoptosis was detected using a fluorescein isothiocyanate (FITC)-Annexin V/Propidium Iodide (PI) Apoptosis Detection Kit (Vazyme Biotech). Briefly, cells from different treatment groups were trypsinized and resuspended in 100 μL of binding buffer containing FITC-Annexin V/PI for 15 min. Apoptosis was analyzed by flow cytometry with a BD FACSCalibur instrument (BD Biosciences, Franklin Lakes, NJ, USA).

### RNA immunoprecipitation (RIP)

RIP was performed using an EZ-Magna RIP Kit (Millipore) according to the manufacturer’s protocol. Briefly, the whole-cell lysate was incubated with RIP buffer containing magnetic beads conjugated to an anti-Ago2 antibody (Millipore) or anti-m^6^A antibody (Epigentek, Farmingdale, NY, USA) at 4 °C for 6 h. Immunoglobulin G (IgG) was used as the negative control. The beads were washed and were then incubated with 0.1% SDS/0.5 mg/mL proteinase K at 55 °C for 30 min to remove proteins. Finally, the coprecipitated RNAs were evaluated by qRT-PCR.

### Dual-luciferase reporter assay

HEK293T cells were cotransfected with the corresponding plasmids and the miR-328-3p mimic or its negative control using Lipofectamine 2000. After 48 h of transfection, cells were lysed, and luciferase activity was assessed using a Dual-Luciferase Reporter Assay Kit (Promega). Firefly luciferase activity was normalized to Renilla luciferase activity.

### Histological analysis and immunohistochemical (IHC) staining of murine knee joints and human cartilage

Human cartilage sections and murine joint sections were fixed with 4% paraformaldehyde, embedded in paraffin, and sliced into 5-μm sections. Sections were deparaffinized in xylene, hydrated through a graded ethanol series, and stained with safranin-O/fast green (Yeasen). For the IHC assay, sections were treated with the appropriate primary antibodies at 4 °C overnight and incubated with secondary antibodies at 37 °C for 1 h.

### Fluorescence in situ hybridization (FISH)

Primary chondrocytes were fixed with 4% formaldehyde for 10 min and washed with PBS. Then, the cells were permeabilized in 0.5% Triton X-100 at 4 °C for 5 min, washed with PBS, and prehybridized at 37 °C for 30 min before hybridization. Anti-AC008 (RiboBio, Cat. No. lnc1101301), anti-U6 (RiboBio, Cat. No. lnc110101), and anti-18S rRNA (RiboBio, Cat. No. lnc110102) oligodeoxynucleotide probes were used in the hybridization solution for incubation at 37 °C overnight in the dark. The next day, the cells were counterstained with 4′,6-diamidino-2-phenylindole (DAPI, Solarbio, Beijing, China) and imaged using a confocal laser scanning microscope.

### Subcellular fractionation assay

Nuclear and cytoplasmic RNAs from primary chondrocytes were separated using a Cytoplasmic and Nuclear RNA Purification Kit (Norgenbiotek Corporation, Thorold, ON, Canada). The expression level of AC008 in the nucleus and cytoplasm of primary chondrocytes was analyzed by qRT-PCR.

### Mouse model of monosodium iodoacetate (MIA)-induced OA

All animal procedures were approved by the Committee on the Ethics of Animal Experiments of Nanjing Medical University, Nanjing, China. In brief, 10-week-old male C57BL/6 mice were randomly divided into six groups: normal group (*n* = 5); MIA group (*n* = 5); MIA + agomir-NC group (*n* = 5); MIA + agomir-328-3p group (*n* = 5); MIA + AAV-NC group (*n* = 5); and MIA + AAV-AC008 group (*n* = 5). For induction of OA, mice were given an intra-articular injection of MIA (Sigma-Aldrich) in the knee. One week later, agomir-328-3p or negative control agomir (RiboBio) and adeno-associated virus expressing AC008 (AAV-AC008) or negative control virus (GeneChem, Shanghai, China) were injected into the knee joints of mice in the appropriate groups. Treatments were administered at 20 μL per joint per mouse once per week for 5 consecutive weeks. Six weeks later, the mice were euthanized, and the knee joints were collected for safranin-O/fast green staining and IHC analysis. All sections were observed and evaluated by three authors. Histological evaluation of plug cartilage was performed using the modified Mankin score.

### Statistical analysis

The data are presented as the mean ± standard deviation values. Statistical analysis was carried out using GraphPad Prism 8.0 software (GraphPad, San Diego, CA, USA). Unpaired two-tailed Student’s *t* test was used to determine the significance of differences between two groups, while two-way analysis of variance (ANOVA) was used for multiple comparisons. Spearman correlation analysis was used to identify correlations. A *P* value of less than 0.05 was considered statistically significant.

## Results

### AC008 is highly expressed in human osteoarthritic cartilage

**Fig. 1:**
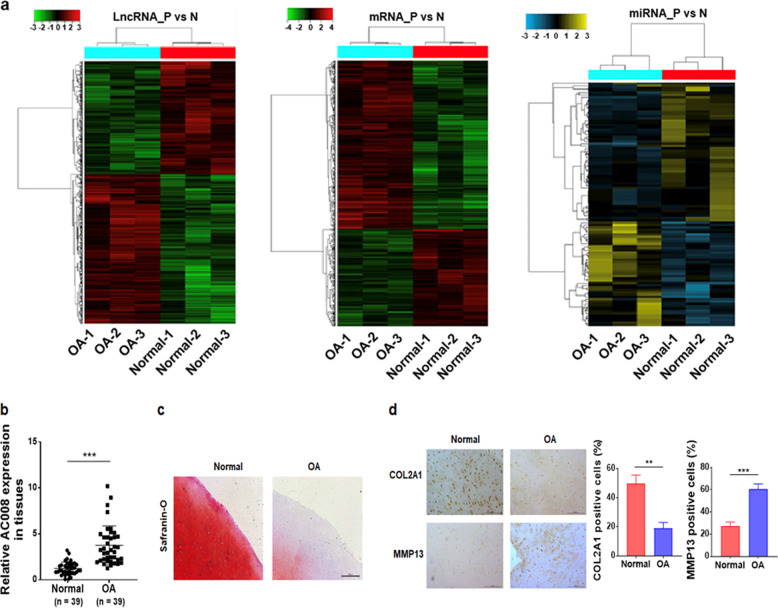
AC008 is upregulated in human osteoarthritic cartilage. **a** A cluster heat map showing the differentially expressed lncRNAs, mRNAs, and miRNAs between normal and osteoarthritic cartilage (*n* = 3). **b** The expression level of AC008 in normal and osteoarthritic cartilage was evaluated by qRT–PCR (*n* = 39). **c** Safranin-O staining was performed to assess ECM degradation in normal and osteoarthritic cartilage samples (scale bar = 500 μm). **d** IHC staining was conducted to evaluate the expression levels of COL2A1 and MMP13 in normal and osteoarthritic cartilage samples (scale bar = 200 μm). The quantification of cells positive for COL2A1 and MMP13 staining is shown in the right panel. OA osteoarthritis. The data are presented as the means ± SDs. Statistical differences were determined by unpaired two-tailed Student’s *t* test (**b**, **d**). ^**^P < 0.01; ^***^P < 0.001.

We performed microarray analyses to identify the differentially expressed lncRNAs, mRNAs, and miRNAs between normal and osteoarthritic articular cartilage. In total, we identified 1194 lncRNAs, 1172 mRNAs, and 105 miRNAs that were differentially expressed between normal and osteoarthritic cartilages (fold change ≥ 2 or ≤ 0.5 and *P* < 0.05; Fig. 1a). Based on the differentially expressed gene data, we performed an integrated analysis to construct the potential lncRNA-miRNA-mRNA network. We found that AC008440.5 (abbreviated AC008), a novel lncRNA, potentially interacted with miR-328-3p. In addition, AQP1 and ANKH, two OA-associated genes^18,19^, were found to be the potential targets of miR-328-3p. Therefore, we selected AC008, miR-328-3p, AQP1, and ANKH for further study. First, we examined the expression of AC008 in a cohort of 39 osteoarthritic cartilage samples. Consistent with the microarray data, AC008 was significantly upregulated in osteoarthritic cartilage samples compared to normal cartilage samples (Fig. 1b). Severe cartilage loss was observed in osteoarthritic cartilage compared to normal cartilage, as indicated by the reduced safranin-O staining (Fig. 1c). In addition, osteoarthritic cartilage displayed decreased expression of COL2A1 and high expression of MMP13, implying ECM degradation (Fig. 1d). These results indicate that AC008 may be involved in the pathogenesis of OA.

### AC008 inhibits chondrocyte viability and promotes chondrocyte apoptosis and ECM degradation

**Fig. 2:**
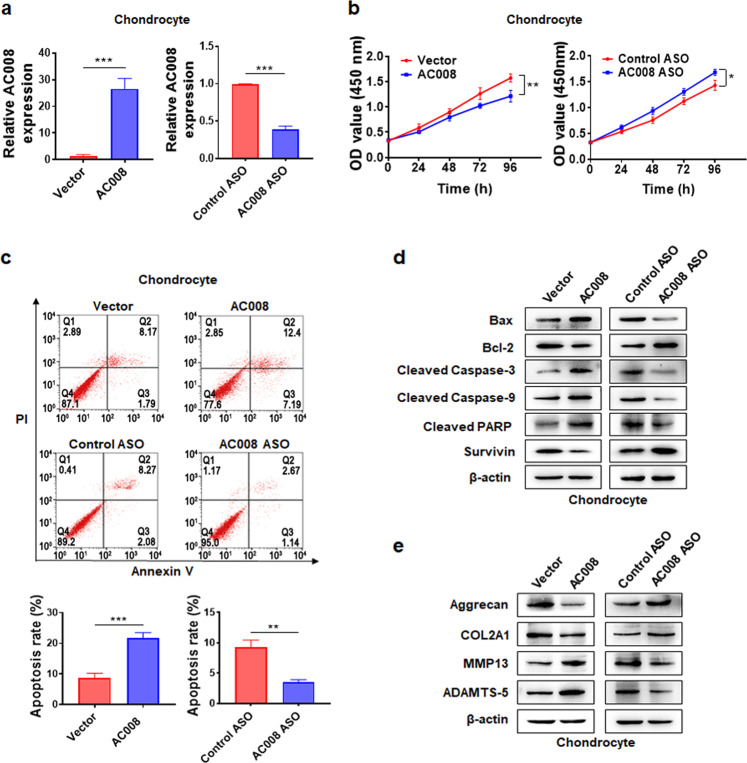
AC008 inhibits chondrocyte viability and promotes chondrocyte apoptosis and ECM degradation. **a** qRT–PCR was performed to verify the overexpression and knockdown efficiencies of AC008 in chondrocytes transfected with pcDNA-AC008 or AC008 ASO. **b** A CCK-8 assay was used to identify the viability of chondrocytes with AC008 overexpression or knockdown. **c** Chondrocyte apoptosis was detected with FITC-Annexin V/PI double staining using flow cytometry following AC008 overexpression or knockdown. **d** The expression levels of apoptosis-associated proteins were evaluated by western blotting. **e** The expression levels of ECM proteins (Aggrecan and COL2A1) and cartilage-degrading enzymes (MMP13 and ADAMTS-5) were analyzed by western blotting in chondrocytes following AC008 overexpression or knockdown. The data are presented as the means ± SDs. Statistical differences were determined using unpaired two-tailed Student’s *t* test (**a**, **c**) or two-way ANOVA (**b**). ^*^P < 0.05; ^**^P < 0.01; ^***^P < 0.001.

To investigate the potential effects of AC008 on the biological activity of chondrocytes, we constructed an AC008 overexpression vector and designed an ASO targeting AC008. qRT-PCR analysis revealed successful overexpression and knockdown of AC008 in primary chondrocytes (Fig. 2a). To exclude the possibility of gDNA or vector DNA contamination in the cDNA, an RT SuperMix kit containing 4× gDNA wiper Mix was used. To further verify the absence of contamination, we performed PCR using primers designed from the pcDNA3.1 vector sequence as well as primers designed from the exon and intron sequences of the GAPDH gene. Agarose gel electrophoresis showed that no PCR products were obtained when cDNA was used as the template. In contrast, PCR products were obtained when gDNA or pcDNA3.1 plasmids were used as templates (Supplementary Fig. 1). These data indicated that there was no gDNA or vector DNA contamination in the cDNA. In addition, the transfection efficiency was monitored by transfection of a GFP-expressing plasmid (Supplementary Fig. 2). The CCK-8 assay demonstrated that AC008 overexpression attenuated chondrocyte viability, whereas AC008 silencing increased chondrocyte viability (Fig. 2b). Next, we assessed the effects of AC008 on chondrocyte apoptosis. Flow cytometric analysis indicated that the apoptosis rate was increased after AC008 overexpression and was reduced following AC008 knockdown (Fig. 2c). In addition, after AC008 overexpression, the expression of antiapoptotic proteins, including Bcl-2 and Survivin, was markedly downregulated, while the expression of proapoptotic proteins, such as Bax, cleaved PARP, cleaved Caspase-3, and cleaved Caspase-9, was upregulated. In contrast, AC008 knockdown resulted in the opposite effects on the expression of these antiapoptotic and proapoptotic markers (Fig. 2d). Since the degradation of articular cartilage is a hallmark of OA pathogenesis, we investigated the impact of AC008 on ECM degradation. AC008 promoted the expression of major cartilage-degrading enzymes, including MMP13 and ADAMTS-5, and inhibited the expression of ECM proteins, such as COL2A1 and Aggrecan, whereas AC008 knockdown exerted the opposite effects on the expression of these genes (Fig. 2e). Taken together, these results suggest that abnormal expression of AC008 might affect OA progression by regulating chondrocyte viability, chondrocyte apoptosis, and ECM degradation.

### AC008 acts as a sponge for miR-328-3p

**Fig. 3:**
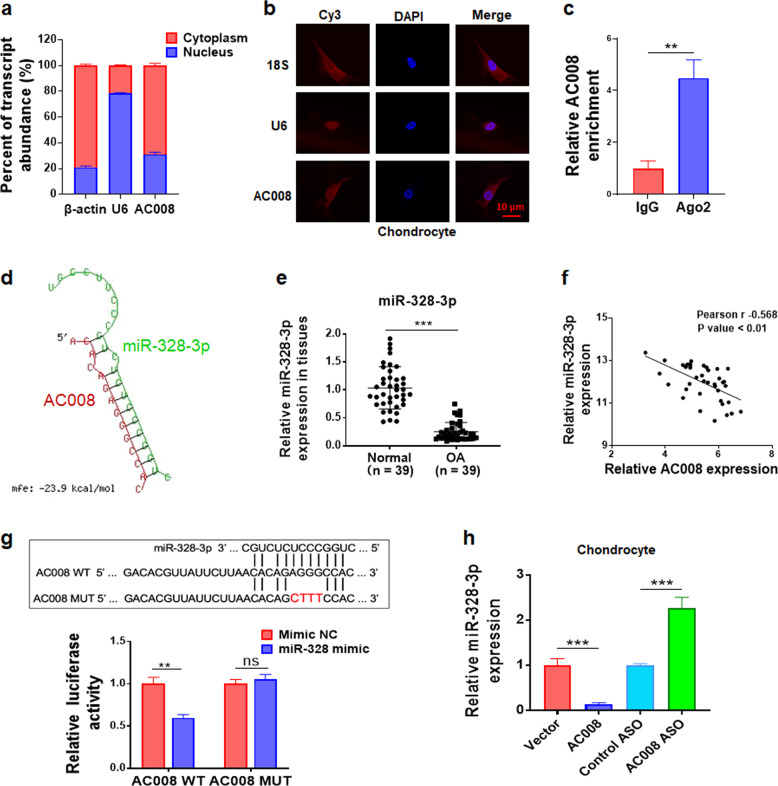
AC008 interacts with miR-328-3p. **a** The expression level of AC008 in the nucleus and cytoplasm of primary chondrocytes was analyzed by qRT-PCR. β-actin and U6 were used as internal controls for the cytoplasmic and nuclear fractions, respectively. **b** FISH was performed to determine the subcellular localization of AC008 in chondrocytes. 18S rRNA and U6 were labeled with cyanine 3 (Cy3, red), and nuclei were stained with DAPI (blue). 18S rRNA and U6 were used as cytoplasmic and nuclear markers, respectively. Scale bar: 10 μm. **c** RIP experiments were performed in primary chondrocytes, and the coprecipitated RNAs were subjected to qRT-PCR to analyze AC008 expression. The fold enrichment of AC008 in the anti-Ago2 precipitate is shown relative to that in the corresponding IgG control precipitate. **d** The potential miR-328-3p binding sites in AC008 were predicted with the RNAhybrid database. **e** The expression level of miR-328-3p in normal and osteoarthritic cartilage was evaluated by qRT-PCR (*n* = 39). **f** The association between AC008 and miR-328-3p expression in osteoarthritic cartilage was assessed by Spearman correlation analysis. **g** Top: the designed wild-type AC008 sequence and mutant AC008 sequence (in which only the putative miR-328-3p binding site was mutated). Bottom: luciferase reporter assay in HEK293T cells transfected with psiCHECK2-AC008 (WT or Mut) and the miR-328-3p mimic or mimic control. **h** qRT-PCR was performed to determine the expression level of miR-328-3p in chondrocytes after transfection with pcDNA-AC008 or AC008 ASO. OA osteoarthritis, WT wild-type, MUT mutant, ns no significant difference. The data are presented as the means ± SDs. Statistical differences were determined using unpaired two-tailed Student’s *t* test (**c**, **e**, **g**, **h**) or Spearman correlation analysis (**f**). ^**^P < 0.01; ^***^P < 0.001.

**Fig. 4:**
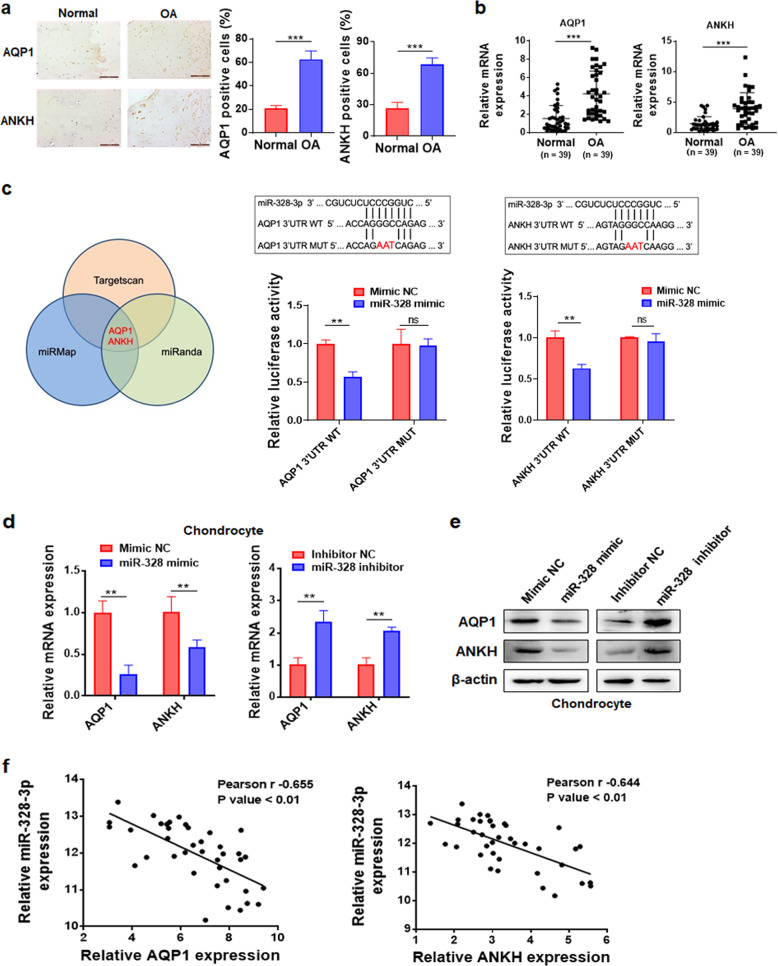
AQP1 and ANKH are direct targets of miR-328-3p. **a** IHC staining was conducted to evaluate the expression levels of AQP1 and ANKH in normal and osteoarthritic cartilage samples (scale bar = 200 μm). The quantification of cells positive for AQP1 and ANKH staining is shown in the right panel. **b** The expression levels of AQP1 and ANKH in normal and osteoarthritic cartilage were evaluated by qRT-PCR (*n* = 39). **c** Left: A Venn diagram showing that AQP1 and ANKH are potential targets of miR-328-3p based on three different target prediction algorithms (miRmap, TargetScan, and miRanda). Right: The putative miR-328-3p binding sites in the 3′UTRs of AQP1 and ANKH and the designed WT/MUT sequences (top); luciferase reporter assay in HEK293T cells transfected with pGL3-AQP1-3′UTR (WT or MUT), pGL3-ANKH-3′UTR (WT or MUT), and the miR-328-3p mimic or its control (bottom). **d**, **e** qRT-PCR (**d**) and western blot analysis (**e**) were performed to evaluate the mRNA and protein levels of AQP1 and ANKH in chondrocytes after transfection with the miR-328-3p mimic, the miR-328-3p inhibitor, or their respective controls. **f** Associations between miR-328-3p and AQP1 or ANKH expression in osteoarthritic cartilage were assessed by Spearman correlation analysis. OA osteoarthritis, WT wild-type, MUT mutant, ns no significant difference. The data are presented as the means ± SDs. Statistical differences were determined using unpaired two-tailed Student’s *t* test (**a**–**d**) or Spearman correlation analysis (**f**). ^**^P < 0.01; ^***^P < 0.001.

To explore the mechanism of action of AC008 in OA, we initially determined the subcellular localization of AC008 in chondrocytes. qRT-PCR analysis showed that AC008 was predominantly localized in the cytoplasm of primary chondrocytes (Fig. 3a), consistent with the results of FISH (Fig. 3b). Given the cytoplasmic distribution of AC008, we hypothesized that AC008 might exert its effects by acting as a ceRNA. Argonaute 2 (Ago2) is a core component of the RNA-induced silencing complex (RISC), which is involved in miRNA-mediated mRNA destabilization or translational repression^20^. To confirm whether AC008 is included in the RISC complex, we performed RIP using an anti-Ago2 antibody. As shown in Fig. 3c, endogenous AC008 was preferentially enriched in the anti-Ago2 immunoprecipitates compared with the IgG immunoprecipitates, suggesting that AC008 might directly bind to miRNAs and function as a ceRNA. By using the LncBase v2 database, we identified 17 miRNAs that may potentially interact with AC008 (Supplementary Table 6). Among these 17 miRNAs, miR-328-3p was significantly downregulated in osteoarthritic cartilage compared to normal cartilage, based on our microarray data. RNAhybrid database analysis further revealed the potential interaction between miR-328-3p and AC008 (Fig. 3d). We selected miR-328-3p for further study, as the expression of miR-328-3p was notably lower in osteoarthritic cartilage than in normal cartilage and was negatively correlated with AC008 expression in osteoarthritic cartilage (Fig. 3e, f). In addition, two OA-associated genes, AQP1 and ANKH, which were identified as upregulated genes by microarray analysis, were potential targets for miR-328-3p (Fig. 4c). Furthermore, the dual-luciferase reporter assay revealed that overexpression of miR-328-3p markedly reduced the luciferase activity of the wild-type AC008 reporter gene but not that of the AC008 mutant vector (Fig. 3g), indicating direct binding between AC008 and miR-328-3p. In addition, we found that overexpression of AC008 dramatically decreased miR-328-3p expression; in contrast, knockdown of AC008 markedly increased miR-328-3p expression (Fig. 3h). These results indicate that AC008 directly sponges miR-328-3p.

We further performed miR-328-3p overexpression and inhibition experiments to determine the function of miR-328-3p in primary chondrocytes (Supplementary Fig. 3a). The CCK-8 assay and flow cytometric analysis demonstrated that miR-328-3p overexpression increased chondrocyte viability (Supplementary Fig. 3b) and attenuated chondrocyte apoptosis (Supplementary Fig. 3c). After miR-328-3p overexpression, the expression levels of Bcl-2 and Survivin were increased, while the expression levels of Bax, cleaved PARP, cleaved Caspase-3, and cleaved Caspase-9 were reduced (Supplementary Fig. 3d). Moreover, miR-328-3p promoted the expression of COL2A1 and Aggrecan and decreased the expression of MMP13 and ADAMTS-5 (Supplementary Fig. 3e). The opposite effect was observed following inhibition of miR-328-3p (Supplementary Fig. 3b–e).

### AQP1 and ANKH are direct targets of miR-328-3p and are able to reverse the effects of miR-328-3p on chondrocyte viability, chondrocyte apoptosis, and ECM degradation

In our microarray data, we identified a total of 1172 differentially expressed mRNAs between normal and osteoarthritic cartilage, among which 739 mRNAs were upregulated and 433 mRNAs were downregulated in osteoarthritic cartilage compared to normal cartilage (Fig. 1a). Two OA-associated genes, AQP1 and ANKH, attracted our attention because they are not only upregulated in osteoarthritic cartilage (Fig. 4a, b) but also potential targets of miR-328-3p, based on bioinformatics analysis (Fig. 4c, left). The direct interaction between miR-328-3p and AQP1 or ANKH was further validated by a dual-luciferase reporter assay (Fig. 4c, right). In addition, the miR-328-3p mimic significantly reduced AQP1 and ANKH expression, while the miR-328-3p inhibitor increased the expression of AQP1 and ANKH (Fig. 4d, e). Moreover, the expression levels of AQP1 and ANKH were negatively correlated with that of miR-328-3p in osteoarthritic cartilage (Fig. 4f).

**Fig. 5:**
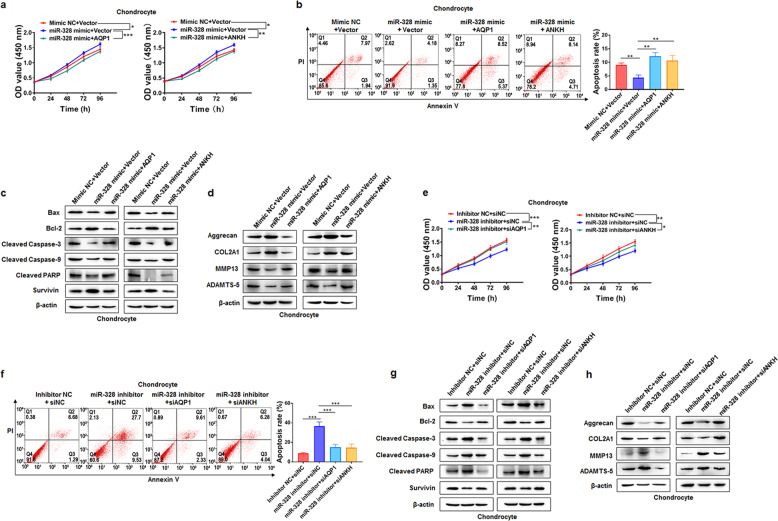
AQP1 and ANKH reverse the effects of miR-328-3p on chondrocyte viability, chondrocyte apoptosis, and ECM degradation. **a** A CCK-8 assay was used to evaluate the viability of chondrocytes transfected with Mimic NC + Vector, miR-328-3p mimic + Vector, miR-328-3p mimic + AQP1, or miR-328-3p mimic + ANKH. **b**, **c** The apoptosis rates (**b**) and the expression levels of apoptosis-associated proteins (**c**) were evaluated in chondrocytes after transfection with Mimic NC + Vector, miR-328-3p mimic + Vector, miR-328-3p mimic + AQP1, or miR-328-3p mimic + ANKH. **d** The expression levels of ECM proteins (Aggrecan and COL2A1) and cartilage-degrading enzymes (MMP13 and ADAMTS-5) were analyzed by western blotting in chondrocytes after transfection with Mimic NC + Vector, miR-328-3p mimic + Vector, miR-328-3p mimic + AQP1, or miR-328-3p mimic + ANKH. **e** A CCK-8 assay was used to evaluate the viability of chondrocytes transfected with Inhibitor NC + siNC, miR-328-3p inhibitor + siNC, miR-328-3p inhibitor + siAQP1, or miR-328-3p inhibitor + siANKH. **f**, **g** The apoptosis rates (**f**) and the expression levels of apoptosis-associated proteins (**g**) were evaluated in chondrocytes after transfection with Inhibitor NC + siNC, miR-328-3p inhibitor + siNC, miR-328-3p inhibitor + siAQP1, or miR-328-3p inhibitor + siANKH. **h** The expression levels of ECM proteins (Aggrecan and COL2A1) and cartilage-degrading enzymes (MMP13 and ADAMTS-5) were analyzed by western blotting in chondrocytes after transfection with Inhibitor NC + siNC, miR-328-3p inhibitor + siNC, miR-328-3p inhibitor + siAQP1, or miR-328-3p inhibitor + siANKH. The data are presented as the means ± SDs. Statistical differences were determined using two-way ANOVA (**a**, **e**) or unpaired two-tailed Student’s *t* test (**b**, **f**). ^*^P < 0.05; ^**^P < 0.01; ^***^P < 0.001.

Although AQP1 and ANKH have been demonstrated to participate in OA progression^21,22^, their roles in chondrocyte viability, chondrocyte apoptosis, and ECM degradation appeared to be minimal. We then overexpressed and inhibited AQP1 and ANKH in primary chondrocytes (Supplementary Fig. 4a). Forced expression of AQP1 and ANKH attenuated chondrocyte viability (Supplementary Fig. 4b), induced chondrocyte apoptosis (Supplementary Fig. 4c, d), decreased COL2A1 and Aggrecan expression, and increased MMP13 and ADAMTS-5 expression (Supplementary Fig. 4e). Opposite alterations were observed in the AQP1 and ANKH knockdown groups (Supplementary Fig. 4b-e). Additionally, overexpression of AQP1 or ANKH partially reversed the effects of miR-328-3p on chondrocyte viability, chondrocyte apoptosis, and ECM degradation (Fig. 5a–d); however, silencing AQP1 or ANKH partially abolished the impacts of the miR-328-3p inhibitor on chondrocyte viability, chondrocyte apoptosis, and ECM degradation (Fig. 5e–h).

### AC008 accelerates OA progression via the miR-328-3p‒AQP1/ANKH axis

**Fig. 6:**
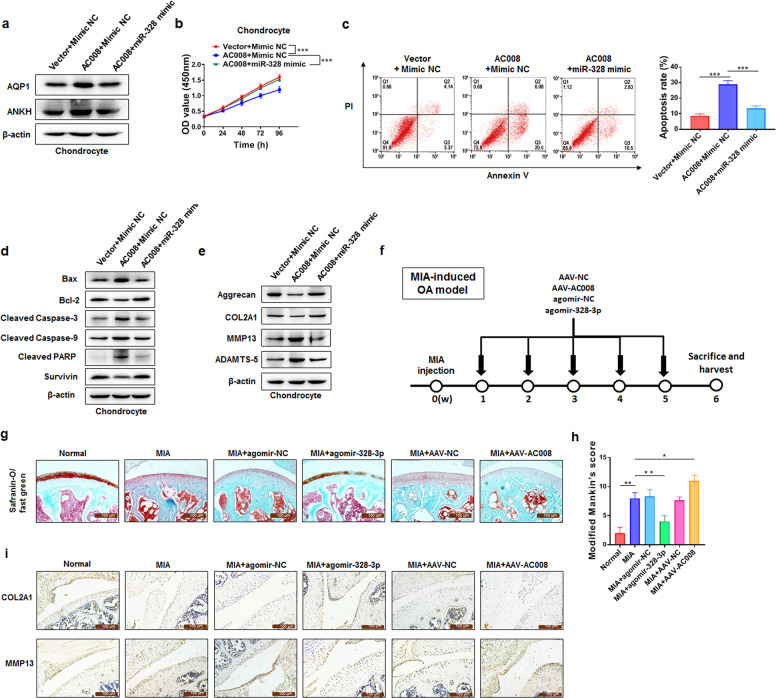
AC008 accelerates OA progression through the miR-328-3p‒AQP1/ANKH axis. **a** Western blotting was performed to evaluate the protein levels of AQP1 and ANKH in chondrocytes after transfection with Vector + Mimic NC, AC008 + Mimic NC, or AC008 + miR-328-3p mimic. **b** A CCK-8 assay was used to evaluate the viability of chondrocytes transfected with Vector + Mimic NC, AC008 + Mimic NC, or AC008 + miR-328-3p mimic. **c**, **d** The apoptosis rates (**c**) and the expression levels of apoptosis-associated proteins (**d**) were evaluated in chondrocytes after transfection with Vector+Mimic NC, AC008 + Mimic NC, or AC008 + miR-328-3p mimic. **e** The expression levels of ECM proteins (Aggrecan and COL2A1) and cartilage-degrading enzymes (MMP13 and ADAMTS-5) were analyzed by western blotting in chondrocytes after transfection with Vector + Mimic NC, AC008 + Mimic NC, or AC008 + miR-328-3p mimic. **f** A schematic diagram illustrating the experimental design for the timeline of intra-articular injection of agomir-NC, agomir-328-3p, AAV-NC, and AAV-AC008 in vivo. **g** Histological sections of cartilage from mice in each group were stained with safranin-O/fast green (representative images; scale bar = 100 μm). **h** The modified Mankin scoring system was used to evaluate articular cartilage in mice. **i** IHC staining was conducted to detect the expression of COL2A1 and MMP13 in cartilage samples harvested from each group (scale bar = 100 μm). MIA monosodium iodoacetate, AAV adeno-associated virus, NC negative control. The data are presented as the means ± SDs. Statistical differences were determined using two-way ANOVA (**b**) or unpaired two-tailed Student’s *t* test (**c**, **h**). ^*^P < 0.05; ^**^P < 0.01; ^***^P < 0.001.

Considering the regulatory relationship between AC008 and miR-328-3p as well as that between miR-328-3p and AQP1/ANKH, we hypothesized that AC008 might modulate AQP1 and ANKH expression by acting as a sponge for miR-328-3p. Indeed, overexpression of AC008 increased the expression of AQP1 and ANKH, and this increase in AQP1 and ANKH expression was partially abolished by the miR-328-3p mimic (Fig. 6a). Moreover, the miR-328-3p mimic reversed the attenuating effect of AC008 on chondrocyte viability (Fig. 6b) and abrogated the proapoptotic effect of AC008 (Fig. 6c, d). In addition, the miR-328-3p mimic reversed the decreases in COL2A1 and Aggrecan expression and abolished the increases in MMP13 and ADAMTS-5 expression induced by AC008 overexpression (Fig. 6e). Taken together, these data indicate that AC008 promotes OA progression by modulating the miR-328-3p‒AQP1/ANKH axis.

Since mouse-derived and human-derived mature miR-328-3p have the same sequence, we used MIA-induced OA mice given treatments with agomir-328-3p, AAV-AC008 and their corresponding control constructs (Fig. 6f). The in vivo delivery efficiencies of agomir-328-3p and AAV-AC008 were validated by qRT–PCR. In addition, the expression levels of AQP1 and ANKH in knee cartilage from MIA + AAV-AC008 mice were higher than those in knee cartilage from MIA + AAV-NC mice but were lower in knee cartilage from MIA + agomir-328-3p mice than in knee cartilage from MIA + agomir-NC mice (Supplementary Fig. 5). Intra-articular injection of MIA induced ECM degradation that resembled the pathological changes of OA, as evidenced by the reduced safranin-O/fast green staining, increased modified Mankin score, decreased expression of COL2A1, and high expression of MMP13, whereas treatment with agomir-328-3p ameliorated MIA-induced OA. Agomir-328-3p effectively reversed the loss of COL2A1 expression and the increase in MMP13 expression in the MIA model. However, treatment with AAV-AC008 resulted in more cartilage destruction than observed in the control group (AAV-NC) of mice with MIA-induced OA (Fig. 6g–i). Thus, consistent with our in vitro findings, these results demonstrated that the AC008‒miR-328-3p‒AQP1/ANKH axis mediates OA progression, suggesting that AC008 may be a promising therapeutic target for OA.

### FTO-dependent m^6^A demethylation mediates the upregulation of AC008 in OA

**Fig. 7:**
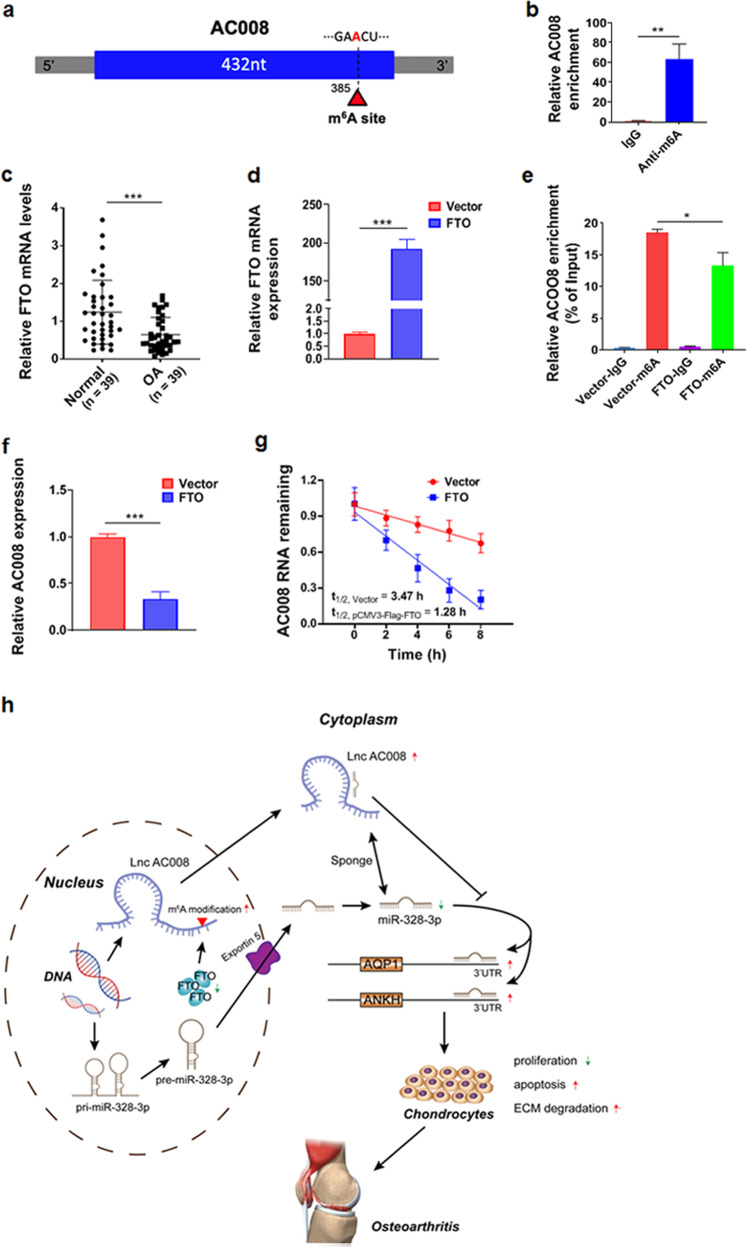
m^6^A modification is involved in the upregulation of AC008 in OA. **a** The m^6^A modification site in AC008 was predicted with the online tool SRAMP (http://www.cuilab.cn/sramp). **b** The m^6^A level of AC008 in chondrocytes was determined by MeRIP-qPCR. **c** The expression level of FTO in normal and osteoarthritic cartilages was evaluated by qRT-PCR (*n* = 39). **d** qRT-PCR was performed to verify the overexpression efficiency of FTO in chondrocytes transfected with the FTO overexpression plasmid. **e** The m^6^A level of AC008 was evaluated in chondrocytes transfected with the indicated plasmids. **f** The expression level of AC008 was evaluated in chondrocytes transfected with the indicated plasmids. **g** Cells were treated with actinomycin D for the indicated times, and qRT-PCR was performed to evaluate the expression level of AC008. **h** Proposed model of the mechanism underlying the expression and function of AC008 in OA progression. The data are presented as the means ± SDs. Statistical differences were determined using unpaired two-tailed Student’s *t* test (**b**–**f**). ^*^P < 0.05; ^**^P < 0.01; ^***^P < 0.001.

Recent studies have suggested that m^6^A modification modulates all phases of RNA metabolism, including RNA folding, stability, splicing, nuclear export, translational modulation, and degradation^23,24^. Therefore, we sought to determine whether m^6^A participates in the upregulation of AC008 in OA. According to the results from the online bioinformatics tool SRAMP (http://www.cuilab.cn/sramp/)^25^, we found an RRACU m^6^A sequence motif in the second exon of AC008 RNA (from position 383–387, Fig. 7a). MeRIP-qPCR showed that m^6^A was highly enriched within the AC008 sequence in chondrocytes (Fig. 7b). FTO is a key m^6^A demethylase (“eraser”) in mammalian cells and has been reported to be associated with OA^26^. Further analysis demonstrated that FTO was significantly downregulated in osteoarthritic cartilage compared to normal cartilage (Fig. 7c). To determine whether FTO-mediated m^6^A demethylation is involved in AC008 upregulation in OA, we overexpressed FTO in chondrocytes (Fig. 7d) and found that the m^6^A level of AC008 was decreased (Fig. 7e). In addition, FTO overexpression led to a decrease of approximately 70% in the expression level of AC008 (Fig. 7f). Furthermore, we treated chondrocytes with actinomycin D to block new RNA synthesis and found that AC008 RNA showed lower stability after overexpression of FTO (Fig. 7g). These findings suggest that higher FTO expression in primary chondrocytes results in removal of m^6^A from AC008, leading to AC008 RNA instability, whereas the lower FTO expression in OA improves AC008 RNA stability, thus contributing to the upregulation of AC008 in OA.

## Discussion

Since lncRNAs play critical roles in the development of cartilage, they are recognized as novel biomarkers for OA diagnosis and prognosis^12,27,28^. In addition, several lncRNAs, such as MEG3^29^ and H19^30^, are considered therapeutic targets for OA. In the present study, we investigated the lncRNA expression profiles of normal and osteoarthritic cartilage using microarray analysis. We focused on the role and potential mechanism of a novel lncRNA, termed AC008, which was aberrantly upregulated in osteoarthritic cartilage. Functionally, we demonstrated that AC008 overexpression decreased chondrocyte viability and induced chondrocyte apoptosis and ECM degradation in vitro and that knockdown of AC008 exerted the opposite effects. Consistent with our in vitro findings, AC008 overexpression induced cartilage matrix loss and accelerated OA progression in a model of MIA-induced OA.

As perturbation of antisense RNA transcription can directly or indirectly regulate the expression of sense genes^31,32^, we also investigated the effect of AC008 on the expression of its coding counterpart, MYADM. Overexpression of AC008 had no obvious effect on MYADM mRNA expression (Supplementary Fig. 6). The cellular localization of lncRNAs is important for their biological functions. Generally, cytoplasmic lncRNAs participate in posttranscriptional regulation by interacting with miRNAs or RNAs, whereas nuclear lncRNAs mediate RNA processing, transcriptional regulation, and chromatin interactions^33,34^. Herein, we identified that AC008 was mainly localized in the cytoplasm and could interact with Ago2, suggesting that AC008 may function as an endogenous miRNA sponge. Bioinformatics analyses and luciferase reporter assays revealed that AC008 interacted with miR-328-3p. In addition, AC008 negatively regulated miR-328-3p expression. However, the pri-miR-328-3p level was not obviously affected by AC008 overexpression (Supplementary Fig. 7), suggesting that AC008 regulates the expression of miR-328-3p but not at the transcriptional level. We speculated that AC008 may regulate miR-328-3p expression by influencing the processing, transport, and/or stability of miR-328-3p.

To date, miR-328-3p has been reported to play important roles in several types of tumors^35,36^, but the role of miR-328-3p in OA is poorly understood. Our data revealed that ectopic miR-328-3p expression increased chondrocyte viability, suppressed chondrocyte apoptosis, and attenuated ECM degradation, whereas inhibition of miR-328-3p exerted the opposite effects. In addition, our experiments in the mouse model of MIA-induced OA further verified the role of miR-328-3p in suppressing the development of OA in vivo.

LncRNAs serve as miRNA sponges to regulate the expression of miRNA target genes^37,38^. According to bioinformatics analysis, miR-328-3p may bind to the 3’UTRs of the AQP1 and ANKH genes; we confirmed these direct associations by dual-luciferase reporter assays. AQP1 is a member of the AQP family and is abundantly expressed in the synovium in rheumatoid arthritis^18,39^. Abnormal expression of AQP1 is closely related to chondrocyte apoptosis and ECM degradation^40,41^. ANKH is widely expressed in multiple tissue types, and dysfunction of ANKH may lead to abnormal mineralization^19^. Consistent with these reports, our study demonstrated that overexpression of AQP1 or ANKH attenuated chondrocyte viability, induced chondrocyte apoptosis, and increased ECM degradation. In contrast, the knockdown of AQP1 or ANKH resulted in the opposite effects. In addition, overexpression of AQP1 or ANKH partially reversed the effects of miR-328-3p on chondrocyte viability, chondrocyte apoptosis, and ECM degradation, whereas knockdown of AQP1 or ANKH partially abolished the effects of the miR-328-3p inhibitor on chondrocyte viability, chondrocyte apoptosis, and ECM degradation. More importantly, the miR-328-3p mimic effectively reversed the AC008-induced increases in AQP1 and ANKH expression. Additionally, the miR-328-3p mimic reversed the attenuating effect of AC008 on viability and abrogated the AC008-induced chondrocyte apoptosis and ECM degradation. These data suggest that AC008 functions as a ceRNA by sponging miR-328-3p, thereby indirectly inhibiting the effects of miR-328-3p on AQP1 and ANKH expression.

Recently, m^6^A modification has been identified as the most abundant internal epitranscriptomic modification in eukaryotic messenger RNAs^42^. M^6^A is installed by m^6^A methyltransferases (also called “writers”, such as METTL3, METTL14, and WTAP), removed by m^6^A demethylases (also called “erasers”, such as FTO and ALKBH5), and recognized by specific RNA-binding proteins (also called “readers”, such as YTHDF1/2/3, eIF3, IGF2BP1/2/3, and HNRNPA2B1)^43^. Although m^6^A modification plays critical roles in a variety of diseases^44–47^, including arthritis^48,49^, studies on m^6^A modification of lncRNAs are scarce in the field of OA. Our data revealed that m^6^A was enriched within the AC008 sequence and that FTO-mediated m^6^A demethylation led to an increase in the instability of AC008 RNA. FTO was initially identified to be associated with obesity and type II diabetes^50,51^. Jia et al.^52^ discovered that FTO binds to m^6^A motifs and removes m^6^A in vitro and in vivo, providing the first example of reversible methylation in RNA. Thus, the lower FTO expression may partially account for the upregulation of AC008 in OA.

In this study, we investigated the biological actions of AC008 on chondrocyte viability, chondrocyte apoptosis, and ECM degradation, and we identified a ceRNA regulatory network, i.e., the AC008‒miR-328-3p‒AQP1/ANKH axis, in OA. We also found that m^6^A modification of AC008 accounts for the upregulation of AC008 in OA (Fig. 7h). Our study reveals that AC008 plays an important role in OA progression and highlights the potential of AC008 as a therapeutic target for OA.

## Supplementary information

Supplementary materials

## Data Availability

The datasets used or analyzed during the current study are available from the corresponding author on reasonable request.

## References

[1] Loeser RF, Goldring SR, Scanzello CR, Goldring MB (2012). Osteoarthritis: a disease of the joint as an organ. Arthritis Rheum..

[2] Glyn-Jones S (2015). Osteoarthritis. Lancet.

[3] Wang X, Hunter D, Xu J, Ding C (2015). Metabolic triggered inflammation in osteoarthritis. Osteoarthr. Cartil..

[4] Hwang HS, Kim HA (2015). Chondrocyte apoptosis in the pathogenesis of osteoarthritis. Int. J. Mol. Sci..

[5] Safiri S (2020). Global, regional and national burden of osteoarthritis 1990-2017: a systematic analysis of the Global Burden of Disease Study 2017. Ann. Rheum. Dis..

[6] Hunter DJ, Bierma-Zeinstra S (2019). Osteoarthritis. Lancet.

[7] Nam JW, Choi SW, You BH (2016). Incredible RNA: dual functions of coding and noncoding. Mol. Cells.

[8] Eidem TM, Kugel JF, Goodrich JA (2016). Noncoding RNAs: regulators of the mammalian transcription machinery. J. Mol. Biol..

[9] Bartel DP (2004). MicroRNAs: genomics, biogenesis, mechanism, and function. Cell.

[10] Huang J (2019). The microRNAs miR-204 and miR-211 maintain joint homeostasis and protect against osteoarthritis progression. Nat. Commun..

[11] Ji ML (2021). Precise targeting of miR-141/200c cluster in chondrocytes attenuates osteoarthritis development. Ann. Rheum. Dis..

[12] Tu J, Huang W, Zhang W, Mei J, Zhu C (2020). The emerging role of lncRNAs in chondrocytes from osteoarthritis patients. Biomed. Pharmacother..

[13] He CP (2021). The function of lncRNAs in the pathogenesis of osteoarthritis. Bone Jt. Res..

[14] Hu J (2018). Long non-coding RNA HOTAIR promotes osteoarthritis progression via miR-17-5p/FUT2/beta-catenin axis. Cell Death Dis..

[15] Liu Y (2020). Long non-coding RNA XIST contributes to osteoarthritis progression via miR-149-5p/DNMT3A axis. Biomed. Pharmacother..

[16] Shi C, Zheng W, Wang J (2021). lncRNA-CRNDE regulates BMSC chondrogenic differentiation and promotes cartilage repair in osteoarthritis through SIRT1/SOX9. Mol. Cell. Biochem..

[17] Bao Q (2019). alphaB-crystallin (CRYAB) regulates the proliferation, apoptosis, synthesis and degradation of extracellular matrix of chondrocytes in osteoarthritis. Exp. Cell Res..

[18] Mu YR, Zhou MY, Cai L, Liu MM, Li R (2020). Overexpression of aquaporin 1 in synovium aggravates rat collagen-induced arthritis through regulating beta-catenin signaling: an in vivo and in vitro study. J. Inflamm. Res..

[19] Williams CJ (2016). The role of ANKH in pathologic mineralization of cartilage. Curr. Opin. Rheumatol..

[20] Tan GS (2009). Expanded RNA-binding activities of mammalian Argonaute 2. Nucleic Acids Res..

[21] Gurley KA, Reimer RJ, Kingsley DM (2006). Biochemical and genetic analysis of ANK in arthritis and bone disease. Am. J. Hum. Genet..

[22] Musumeci G (2013). Aquaporin 1 (AQP1) expression in experimentally induced osteoarthritic knee menisci: an in vivo and in vitro study. Tissue Cell.

[23] Zhao BS, Roundtree IA, He C (2017). Post-transcriptional gene regulation by mRNA modifications. Nat. Rev. Mol. Cell Biol..

[24] Roundtree IA, Evans ME, Pan T, He C (2017). Dynamic RNA modifications in gene expression regulation. Cell.

[25] Zhou Y, Zeng P, Li YH, Zhang Z, Cui Q (2016). SRAMP: prediction of mammalian N6-methyladenosine (m6A) sites based on sequence-derived features. Nucleic Acids Res..

[26] Panoutsopoulou K (2014). The effect of FTO variation on increased osteoarthritis risk is mediated through body mass index: a Mendelian randomisation study. Ann. Rheum. Dis..

[27] Fu M (2015). Expression profile of long noncoding RNAs in cartilage from knee osteoarthritis patients. Osteoarthr. Cartil..

[28] Zhao Y, Xu J (2018). Synovial fluid-derived exosomal lncRNA PCGEM1 as biomarker for the different stages of osteoarthritis. Int. Orthop..

[29] Su, L., Wang, Y., Bao, Y., Liu, X. & Xu, H. LncRNA MEG3 promotes recovery of knee osteoarthritis in rats through regulating VEGF expression. *Panminerva Med*. 10.23736/S0031-0808.19.03832-1 (2020). 10.23736/S0031-0808.19.03832-1 32000463

[30] Tan F, Wang D, Yuan Z (2020). The fibroblast-like synoviocyte derived exosomal long non-coding RNA H19 alleviates osteoarthritis progression through the miR-106b-5p/TIMP2 axis. Inflammation.

[31] Katayama S (2005). Antisense transcription in the mammalian transcriptome. Science.

[32] Rosok O, Sioud M (2004). Systematic identification of sense-antisense transcripts in mammalian cells. Nat. Biotechnol..

[33] Tay Y, Rinn J, Pandolfi PP (2014). The multilayered complexity of ceRNA crosstalk and competition. Nature.

[34] Batista PJ, Chang HY (2013). Long noncoding RNAs: cellular address codes in development and disease. Cell.

[35] Srivastava AK (2019). Inhibition of miR-328-3p impairs cancer stem cell function and prevents metastasis in ovarian cancer. Cancer Res..

[36] Shi J (2019). miR-328-3p mediates the anti-tumor effect in osteosarcoma via directly targeting MMP-16. Cancer Cell Int..

[37] Yu Y, Gao F, He Q, Li G, Ding G (2020). lncRNA UCA1 functions as a ceRNA to promote prostate cancer progression via sponging miR143. Mol. Ther. Nucleic Acids.

[38] Chen X (2020). lncRNA UCA1 promotes gefitinib resistance as a ceRNA to target FOSL2 by sponging miR-143 in non-small cell lung cancer. Mol. Ther. Nucleic Acids.

[39] Mobasheri A, Moskaluk CA, Marples D, Shakibaei M (2010). Expression of aquaporin 1 (AQP1) in human synovitis. Ann. Anat..

[40] Gao H (2016). Aquaporin 1 contributes to chondrocyte apoptosis in a rat model of osteoarthritis. Int. J. Mol. Med..

[41] Haneda M (2018). Depletion of aquaporin 1 decreased ADAMTS4 expression in human chondrocytes. Mol. Med. Rep..

[42] Wang X (2014). N6-methyladenosine-dependent regulation of messenger RNA stability. Nature.

[43] Yang Y, Hsu PJ, Chen YS, Yang YG (2018). Dynamic transcriptomic m(6)A decoration: writers, erasers, readers and functions in RNA metabolism. Cell Res..

[44] Panneerdoss S (2018). Cross-talk among writers, readers, and erasers of m(6)A regulates cancer growth and progression. Sci. Adv..

[45] Weng H (2018). METTL14 inhibits hematopoietic stem/progenitor differentiation and promotes leukemogenesis via mRNA m(6)A modification. Cell Stem Cell.

[46] Deng X (2018). RNA N(6)-methyladenosine modification in cancers: current status and perspectives. Cell Res..

[47] Wu Y (2018). Mettl3-mediated m(6)A RNA methylation regulates the fate of bone marrow mesenchymal stem cells and osteoporosis. Nat. Commun..

[48] Liu Q, Li M, Jiang L, Jiang R, Fu B (2019). METTL3 promotes experimental osteoarthritis development by regulating inflammatory response and apoptosis in chondrocyte. Biochem. Biophys. Res. Commun..

[49] Mo XB, Zhang YH, Lei SF (2018). Genome-wide identification of N(6)-methyladenosine (m(6)A) SNPs associated with rheumatoid arthritis. Front. Genet..

[50] Dina C (2007). Variation in FTO contributes to childhood obesity and severe adult obesity. Nat. Genet..

[51] Scuteri A (2007). Genome-wide association scan shows genetic variants in the FTO gene are associated with obesity-related traits. PLoS Genet..

[52] Jia G (2011). N6-methyladenosine in nuclear RNA is a major substrate of the obesity-associated FTO. Nat. Chem. Biol..

