# Supplementary material for: m6A-mediated upregulation of AC008 promotes osteoarthritis progression through the miR-328-3p‒AQP1/ANKH axis
Source: Exp Mol Med. 2021 Nov 4;53(11):1723–34. doi: 10.1038/s12276-021-00696-7 (PMC8640060; doi:10.1038/s12276-021-00696-7)
Supplement: Supplementary file 1 — Supplementary materials [file 12276_2021_696_MOESM1_ESM.pdf]

Supplementary figures

Supplementary Fig. 1 RNA from chondrocytes transfected with pcDNA 3.1 vector was analyzed by RT–PCR to ensure the complete removal of the genomic or vector DNA. Lane 1 and lane 3: cDNAs were used as templates, and the primers were designed from GAPDH gene sequence (Lane 1) or pcDNA3.1 vector sequence (Lane 3), respectively; lane 2: gDNA was used as a template, and the primers were designed from GAPDH gene sequence; lane 4: pcDNA3.1 plasmids were used as templates, and the primers were designed from pcDNA3.1 vector sequence.

Supplementary Fig. 2 Representative fluorescent images of GFP-positive cells. The primary chondrocytes were transfected with GFP-expressing plasmids, and then analyzed by fluorescence microscopy 48 h after transfection. Scale bar: 200 μm.

Supplementary Fig. 3 miR-328-3p promotes cell viability, and inhibits cell apoptosis and ECM degradation in primary chondrocytes. (a) qRT–PCR assay was performed to verify the overexpression and knockdown efficiencies of miR-328-3p in primary chondrocytes transfected with miR-328-3p mimic, miR-328-3p inhibitor, or the respective controls. (b) A CCK-8 assay was used to evaluate the viability of chondrocytes after transfection with miR-328-3p mimic, miR-328-3p inhibitor, or the respective controls. (c, d) The apoptosis rates (c) and the expression levels of apoptosis-associated proteins (d) were evaluated in primary chondrocytes after transfection with miR-328-3p mimic, miR-328-3p inhibitor, or the respective controls. (e) The expression levels of ECM proteins (Aggrecan and COL2A1) and cartilage-degrading enzymes (MMP13 and ADAMTS-5) were

analyzed by western blotting in primary chondrocytes after transfection with miR-328-3p mimic, miR-328-3p inhibitor, or the respective controls. The data are presented as the means ± SDs. Statistical differences were determined using unpaired two-tailed Student's *t* test (a, c) or two-way ANOVA (b). $^{*}P < 0.05$; $^{***}P < 0.001$.

Supplementary Fig. 4 AQP1 and ANKH inhibit cell viability, and promote cell apoptosis and ECM degradation in primary chondrocytes. (a) qRT–PCR assay was performed to verify the overexpression and knockdown efficiencies of AQP1 and ANKH in primary chondrocytes transfected with AQP1 and ANKH overexpression plasmids, siRNA against AQP1 and ANKH, or the respective controls. (b) A CCK-8 assay was used to evaluate the viability of primary chondrocytes with AQP1 and ANKH overexpression or knockdown. (c, d) The apoptosis rates (c) and the expression levels of apoptosis-related proteins (d) were evaluated in primary chondrocytes with AQP1 and ANKH overexpression or knockdown. (e) The expression levels of ECM proteins (Aggrecan and COL2A1) and cartilage-degrading enzymes (MMP13 and ADAMTS-5) were analyzed by western blotting in primary chondrocytes with AQP1 and ANKH overexpression or knockdown. The data are presented as the means ± SDs. Statistical differences were determined using unpaired two-tailed Student's *t* test (a, c) or two-way ANOVA (b). $^{*}P < 0.05$; $^{**}P < 0.01$; $^{***}P < 0.001$.

Supplementary Fig. 5 qRT–PCR was performed to evaluate the expression levels of AC008, miR-328-3p, AQP1 and ANKH in the knee cartilage from indicated mice (n = 5). The data are presented as the means ± SDs. Statistical differences were

determined by unpaired two-tailed Student's *t* test. **P < 0.01.

Supplementary Fig. 6 The mRNA expression levels of MYADM were determined by qRT–PCR in chondrocytes transfected with pcDNA-AC008 (a), AC008 ASO (b) or the respective controls. The data are presented as the means ± SDs.

Supplementary Fig. 7 qRT–PCR was performed to detect the expression levels of pri-miR-328-3p in AC008-overexpressing chondrocytes. The data are presented as the means ± SDs. Statistical differences were determined by unpaired two-tailed Student's *t* test. $^{***}P < 0.001$. ns, no significant difference.

Supplementary Fig.1

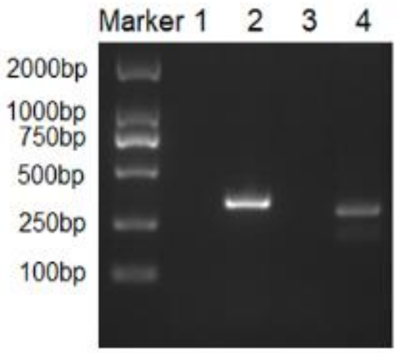

Supplementary Fig. 2

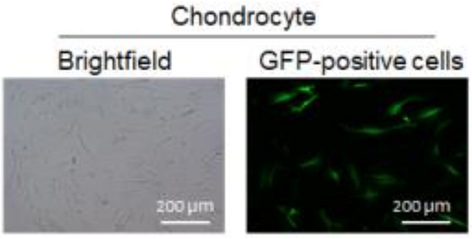

Supplementary Fig. 3

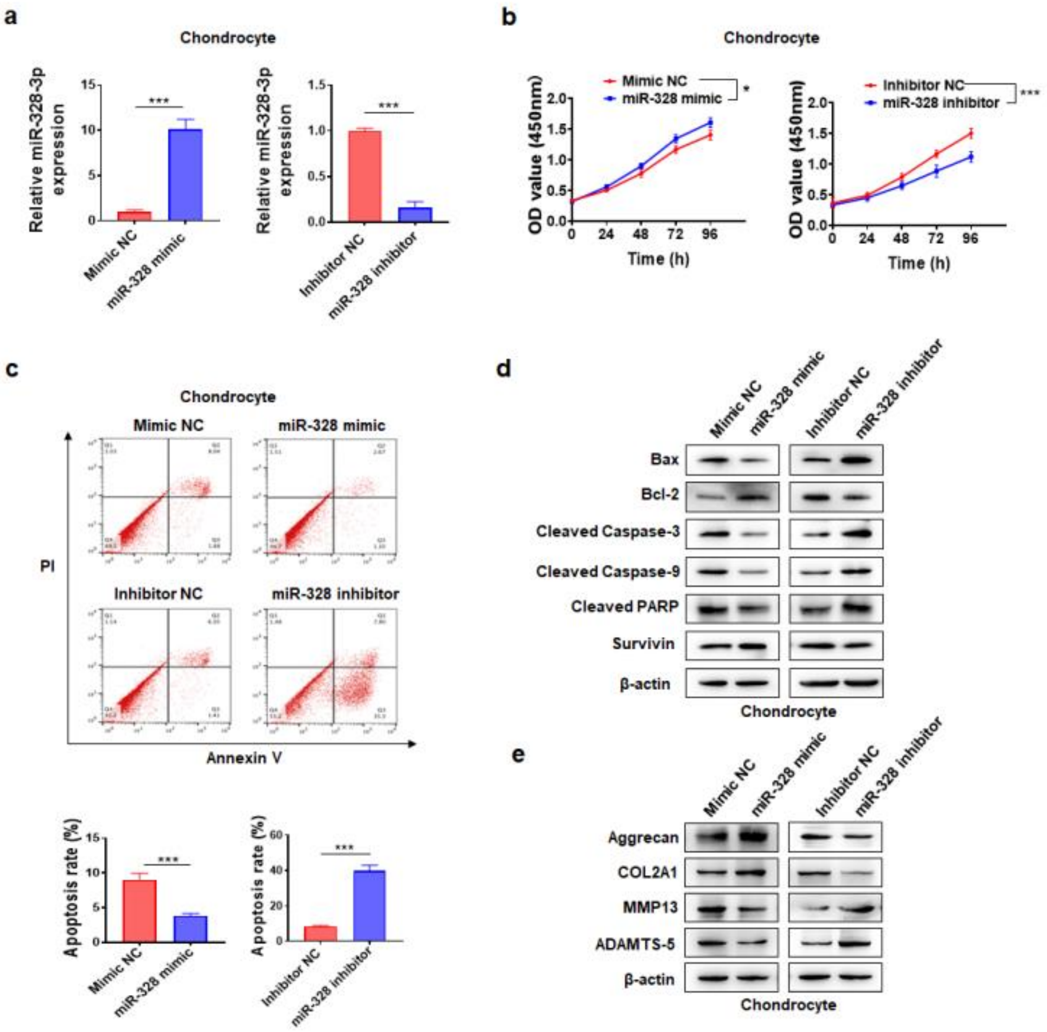

Supplementary Fig. 4

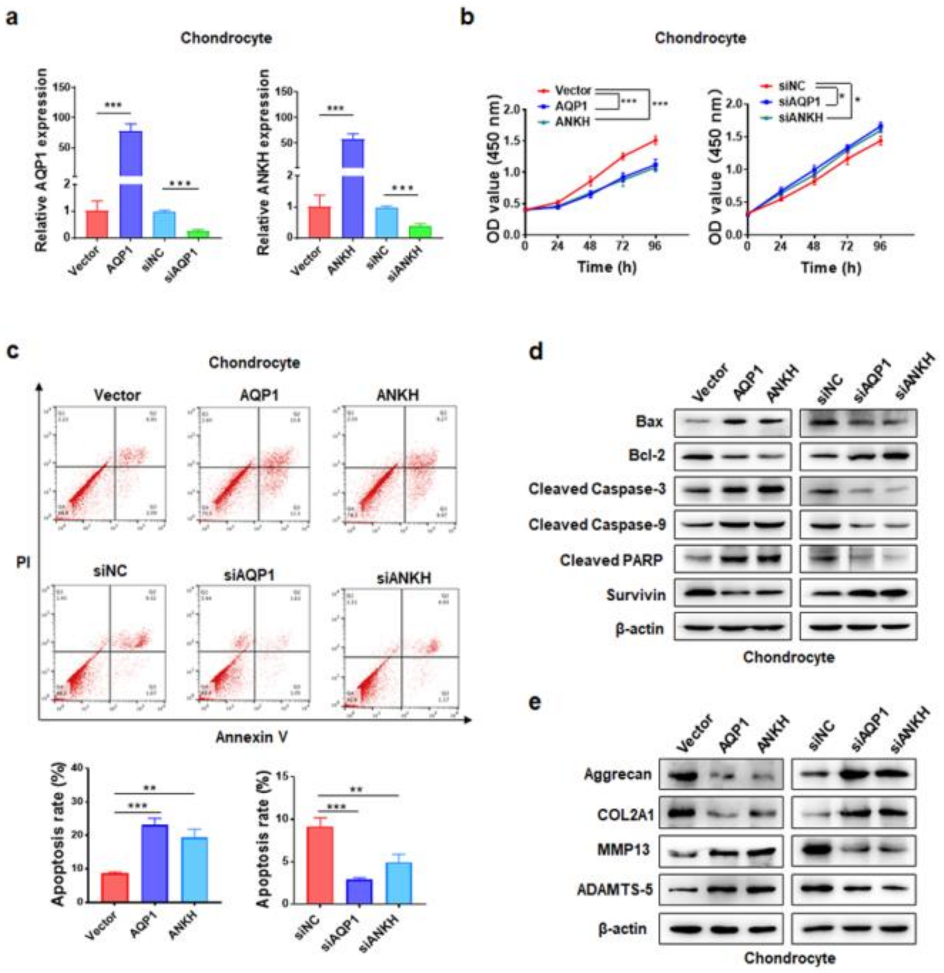

Supplementary Fig. 5

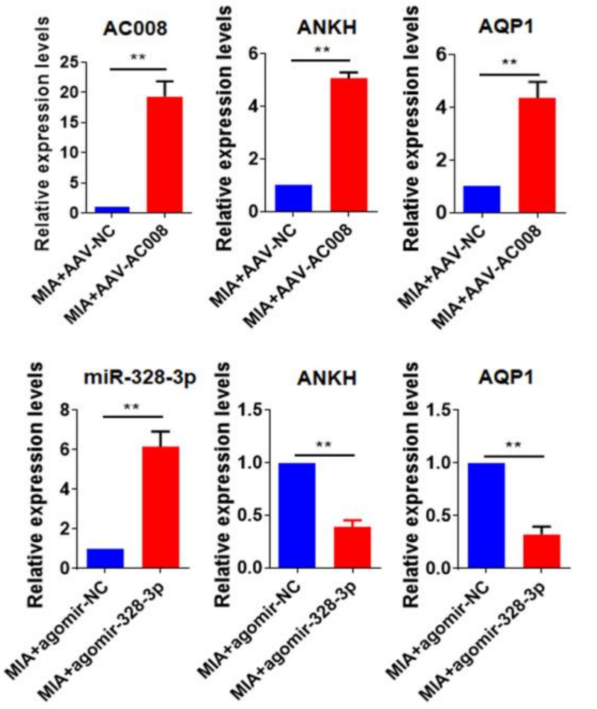

Supplementary Fig. 6

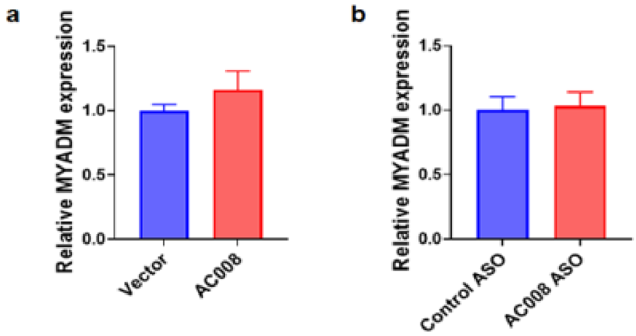

Supplementary Fig. 7

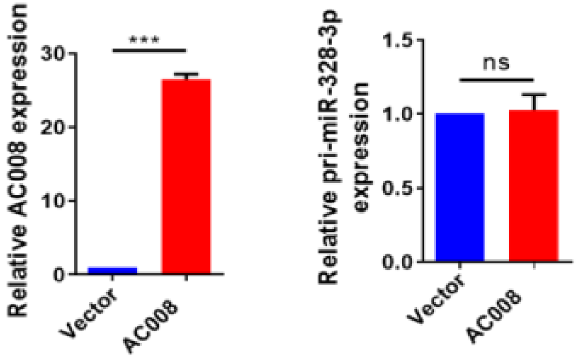

Supplementary tables

Supplementary Table 1 Cartilage donor information

**Normal cartilage tissue**			**OA cartilage tissue**		
**ID**	**Age**	**Gender**	**ID**	**Age**	**Gender**
N1	53	M	P1	58	F
N2	45	F	P2	59	F
N3	83	F	P3	78	M
N4	86	F	P4	59	M
N5	83	F	P5	65	M
N6	91	F	P6	83	F
N7	89	M	P7	49	F
N8	70	M	P8	71	F
N9	84	M	P9	75	M
N10	81	M	P10	56	M
N11	82	F	P11	67	M
N12	64	F	P12	69	M
N13	83	F	P13	61	F
N14	76	M	P14	75	F
N15	69	F	P15	70	F
N16	83	F	P16	73	F
N17	76	F	P17	71	F
N18	88	F	P18	80	F
N19	74	F	P19	71	F
N20	80	F	P20	78	F
N21	75	M	P21	53	F
N22	65	F	P22	67	F
N23	72	F	P23	73	F
N24	78	F	P24	72	F
N25	81	F	P25	76	F

N26	80	F	P26	73	F
N27	79	F	P27	72	F
N28	73	F	P28	68	F
N29	72	F	P29	76	F
N30	91	F	P30	71	F
N31	68	F	P31	68	M
N32	90	F	P32	78	F
N33	81	F	P33	61	F
N34	91	F	P34	62	F
N35	94	F	P35	70	F
N36	84	F	P36	76	F
N37	61	F	P37	71	M
N38	84	M	P38	66	F
N39	84	F	P39	64	F

Supplementary Table 2 Primers used for plasmid constructs

Primer names	Forward/ Reverse	Sequences 5'-3'
pcDNA-AC008	Forward	CAAGCTTGCCAGGAAAAAGAAAGAAAAACAAAAC
	Reverse	CCTCGAGCTTTTTCTTTCCTTCCCAATTCCTTG
pGL3-AQP1-3’UTR-WT	Forward	GCTCTAGAGAGGTAGCTATAGTTGCAG
	Reverse	GCTCTAGAATATGCAGAGCAGACAGAT
pGL3-AQP1-3’UTR-Mut	Forward	CCTTTCAATTCCACCAGAATCAGAGCAGCT
	Reverse	ATTCTGGTGGAATTGAAAGGACTTATGTAGATA
pGL3-ANKH-3’UTR-WT	Forward	GCTCTAGACCACTCACTGTACAACCTC
	Reverse	GCTCTAGACATGGAACAGAATCACGTG
pGL3-ANKH-3’UTR-Mut	Forward	CTGTCTAAAGCTAGTAGAATCAAGGAGTTA
	Reverse	ATTCTACTAGCTTTAGACAGTTCCCTGATC
psiCHECK2-AC008-WT	Forward	CCTCGAGGCCAGGAAAAAGAAAGAAAAACAAAAC
	Reverse	TTGCGGCCGCCTTTTTCTTTCCTTCCCAATTCCTTG
psiCHECK2-AC008-Mut	Forward	TTATTCTTAACACAGCTTTCCACATCGCTG
	Reverse	AAAGCTGTGTTAAGAATAACGTGTCCTGC

Supplementary Table 3 Primers used for qRT–PCR assay

Gene names	Forward/Reverse	Sequences 5'-3'
AQP1	Forward	TGGACACCTCCTGGCTATTG
	Reverse	GGGCCAGGATGAAGTCGTAG
ANKH	Forward	CAAGAGAGACAGGACCAAAGC
	Reverse	TCCACATGGTGCAGTTTATTG
AC008	Forward	AACATCCTGTGGTTCTTCGTCC
	Reverse	TTCCTGTGTTGTTTTGTTGCCC
FTO	Forward	TGGGTTCATCCTACAACGG
	Reverse	CCTCTTCAGGGCCTTCAC
MYADM	Forward	AACATCCTGTGGTTCTTCGTCC
	Reverse	TTCCTGTGTTGTTTTGTTGCCC
Pri-miR-328-3p	Forward	GATGCCCACACAGAAGAGCTC
	Reverse	CCCTGTGAGTGGCCTGGACT
β-actin	Forward	AGATGTGGATCAGCAAGCAG
	Reverse	GCGCAAGTTAGGTTTTGTCA

Supplementary Table 4 Primers used for stem-loop qRT–PCR assay

Gene names	RT/Forward/Reverse	Sequences 5'-3'
miR-328-3p	RT	GTCGTATCCAGTGCAGGGTCCGAGGTATT CGCACTGGATACGACACGGAAGG
	Forward	CTGGCCCTCTCTGCCC
	Reverse	CCAGTGCAGGGTCCGAGG
U6	RT	AACGCTTCACGAATTTGCGT
	Forward	CTCGCTTCGGCAGCACA
	Reverse	AACGCTTCACGAATTTGCGT

Supplementary Table 5 Primers used for PCR assay

Gene names	Forward/Reverse	Sequences 5'-3'
GAPDH (exon-intron)	Forward	GCAAGGAGAGCTCAAGGTCAG
	Reverse	TCCTTCCCGGTTGCAACATGG
pcDNA3.1-backbone	Forward	TAATACGACTCACTATAGGG
	Reverse	CCCCAGAATAGAATGACACC

Supplementary Table 6 MiRNAs that potentially interact with AC008 were predicted by using LncBase v2 database

LncRNA ID	LncRNA	MiRNA	Score
ENSG00000232220	AC008	miR-6734-3p	0.996
ENSG00000232220	AC008	miR-6868-3p	0.956
ENSG00000232220	AC008	miR-627-3p	0.954
ENSG00000232220	AC008	miR-3124-3p	0.909
ENSG00000232220	AC008	miR-4753-3p	0.905
ENSG00000232220	AC008	miR-6875-3p	0.899
ENSG00000232220	AC008	miR-3126-5p	0.895
ENSG00000232220	AC008	miR-6802-3p	0.894
ENSG00000232220	AC008	miR-7703	0.862
ENSG00000232220	AC008	miR-3667-3p	0.842
ENSG00000232220	AC008	miR-6834-5p	0.839
ENSG00000232220	AC008	miR-6875-5p	0.819
ENSG00000232220	AC008	miR-2052	0.816
ENSG00000232220	AC008	miR-4778-3p	0.803
ENSG00000232220	AC008	miR-663b	0.753
ENSG00000232220	AC008	miR-328-3p	0.751
ENSG00000232220	AC008	miR-1294	0.703
